# Effect of Ferric Derisomaltose on Fatigue in Iron Deficiency Anemia Associated With Abnormal Uterine Bleeding

**DOI:** 10.1002/ajh.27555

**Published:** 2024-12-12

**Authors:** Petra Stute, Imo J. Akpan, Christian Breymann, Ally Murji, Sarah H. O'Brien, Jacquelyn M. Powers, Malcolm G. Munro

**Affiliations:** ^1^ Department of Obstetrics and Gynecology Inselspital, Bern University Hospital Bern Switzerland; ^2^ Division of Hematology and Oncology Columbia University Irving Medical Center New York New York USA; ^3^ OBGYN Center Gyn‐Perinatal Zürich Hirslanden Clinics Zürich Switzerland; ^4^ Department of Obstetrics and Gynecology, Trillium Health Partners and The Institute for Better Health University of Toronto Toronto Ontario Canada; ^5^ Division of Pediatric Hematology, Oncology, and Blood and Marrow Transplant Nationwide Children's Hospital and The Ohio State University College of Medicine Columbus Ohio USA; ^6^ Department of Pediatrics, Division of Hematology/Oncology Baylor College of Medicine, Texas Children's Cancer and Hematology Center Houston Texas USA; ^7^ Department of Pediatrics Baylor College of Medicine Houston Texas USA; ^8^ Department of Obstetrics and Gynecology David Geffen School of Medicine at UCLA Los Angeles California USA

To the Editor,

1

Anemia is prevalent among women of reproductive age, with iron deficiency (ID) being the primary etiology. ID can lead to fatigue and reduced quality of life, particularly in the context of abnormal menstrual bleeding [1]. Abnormal menstrual bleeding refers to a set of symptoms collectively known as abnormal uterine bleeding (AUB), and includes heavy menstrual bleeding (HMB), experienced by up to around 50% of reproductive‐aged women [1, 2]. Notably, as many as 63% of women with HMB have been reported to have ID or iron deficiency anemia (IDA) [1]. Despite symptoms of IDA being common alongside AUB, there is a paucity of data on response to intravenous (IV) iron treatment in such a population.

This *post hoc* analysis evaluated pooled data from IV iron‐treated participants with AUB‐associated IDA from two Phase III trials, PROVIDE and FERWON‐IDA (ClinicalTrials.gov identifiers: NCT02130063 and NCT02940886, respectively). The detailed study designs and results of these trials have been published previously [3, 4]. While the trials compared the efficacy and safety of ferric derisomaltose (FDI; a high‐dose formulation) to a low‐dose IV iron, iron sucrose (IS), the main focus of this analysis was the effect of FDI – the newer IV iron associated with increased convenience through its high‐dose regimen – on fatigue, a symptom frequently experienced by women with ID or IDA [1].

Female participants from the FDI treatment arms of PROVIDE and FERWON‐IDA were included in this *post hoc* analysis if they had received treatment, were aged 18–55 years, and had AUB‐associated IDA (defined as hemoglobin [Hb] < 12 g/dL, serum ferritin < 100 ng/mL, and transferrin saturation < 20% at baseline). The underlying cause of IDA was recorded in the trials, but AUB was not a term specified in the case report form; instead, a range of terms were used, including menorrhagia, metrorrhagia, and uterine hemorrhage, which were categorized under AUB in this *post hoc* analysis. Participants with uterine leiomyoma listed as the cause of their IDA were also included in the analysis, as it was assumed that they experienced AUB.

The primary outcome was the change from Week 0 (baseline) to Week 8 (last study visit in FERWON‐IDA) in the Functional Assessment of Chronic Illness Therapy (FACIT) Fatigue Scale score, a 13‐item patient‐reported measure of fatigue and its impact on daily functioning over the past 7 days [5]. Each item is marked from 0 (“not at all”) to 4 (“very much”) on a 5‐point Likert scale [5]. The data are then transformed according to scoring guidelines [5]. The resulting total score falls within the range of 0–52, with a lower score indicating greater fatigue [5]. Fatigue data were collected by both trials.

Other outcomes included change from baseline to Week 8 in Hb, and the proportions of participants with a hematological response (defined as Hb ≥ 12 g/dL or an increase in Hb of ≥ 2 g/dL) and those with a fatigue response at the last study visit in PROVIDE (Week 5) and FERWON‐IDA (Week 8). Two definitions of fatigue response were used: a FACIT Fatigue Scale score increase of ≥ 5 points or an increase of ≥ 12 points at the last study visit in PROVIDE and FERWON‐IDA. These definitions were based on clinically important differences in FACIT Fatigue Scale scores estimated in other populations, ranging from approximately 3 to 5 points [6]. A 12‐point improvement on the FACIT Fatigue Scale corresponds with “much better” patient health in the Physician's Global Assessment of disease activity in individuals with inflammatory bowel disease [5]. Furthermore, the study investigated whether there was a difference in the proportion of participants with or without a hematological response who had experienced a fatigue response following treatment with FDI. Safety was assessed through adverse drug reaction (ADR) reporting.

All data were summarized descriptively, except for the analysis investigating whether participants with or without a hematological response had a fatigue response. For this analysis, *p*‐values were derived from a logistic regression model with treatment and hematological subgroup (i.e., participants with or without a hematological response) as factors and with an interaction between treatment and hematological subgroup; *p*‐values < 0.05 were deemed statistically significant. Responder analyses used observed case data with no missing data imputation. Analyses were performed using SAS software (version 9.4) (SAS Institute Inc., Cary, NC, USA).

Data from 626 participants with AUB‐associated IDA who were treated with FDI were pooled from PROVIDE (*n* = 148) and FERWON‐IDA (*n* = 478) and included. The safety analysis set included all randomized participants who received at least one dose of IV iron and comprised 626 participants. The full analysis set included all randomized participants, according to the planned treatment, who received at least one dose of IV iron and had at least one documented post‐baseline Hb measurement. This set consisted of 620 participants and included one participant randomized to FDI who received IS in error. A flow diagram of participants from PROVIDE and FERWON‐IDA included in the *post hoc* analysis is shown in Appendix 1. Baseline demographics and clinical characteristics of the FDI population are shown in Appendix 2.

The mean (standard deviation [SD]) cumulative dose of FDI in the safety analysis set was 1,139 (385) mg. The median cumulative dose received was 1,000 mg, while the maximum was 2,000 mg. Of the 626 participants in the safety analysis set, 492 (78.6%) received one dose, 133 (21.2%) received two doses, and 1 (0.2%) received three doses.

At baseline, the mean (SD) FACIT Fatigue Scale score was 25.4 (11.9), which is below the mean scores reported for the United States general population, but improved to Week 5 (42.7 [8.6]) and remained substantially higher than baseline at Week 8 (40.9 [9.7]) (Figure 1A). The overall mean (SD) change in FACIT Fatigue Scale score from baseline to Week 8 was 15.15 (12.45). Hb also increased during the study period (Figure 1B). Subgroup analyses were conducted to check for an effect of age, race, severity of anemia, Hb level, number of FDI doses received, and leiomyoma status; no relevant differences were seen in fatigue measures or Hb between subgroups; models did not converge for FACIT Fatigue Scale score stratified by race (data not shown).

**FIGURE 1:**
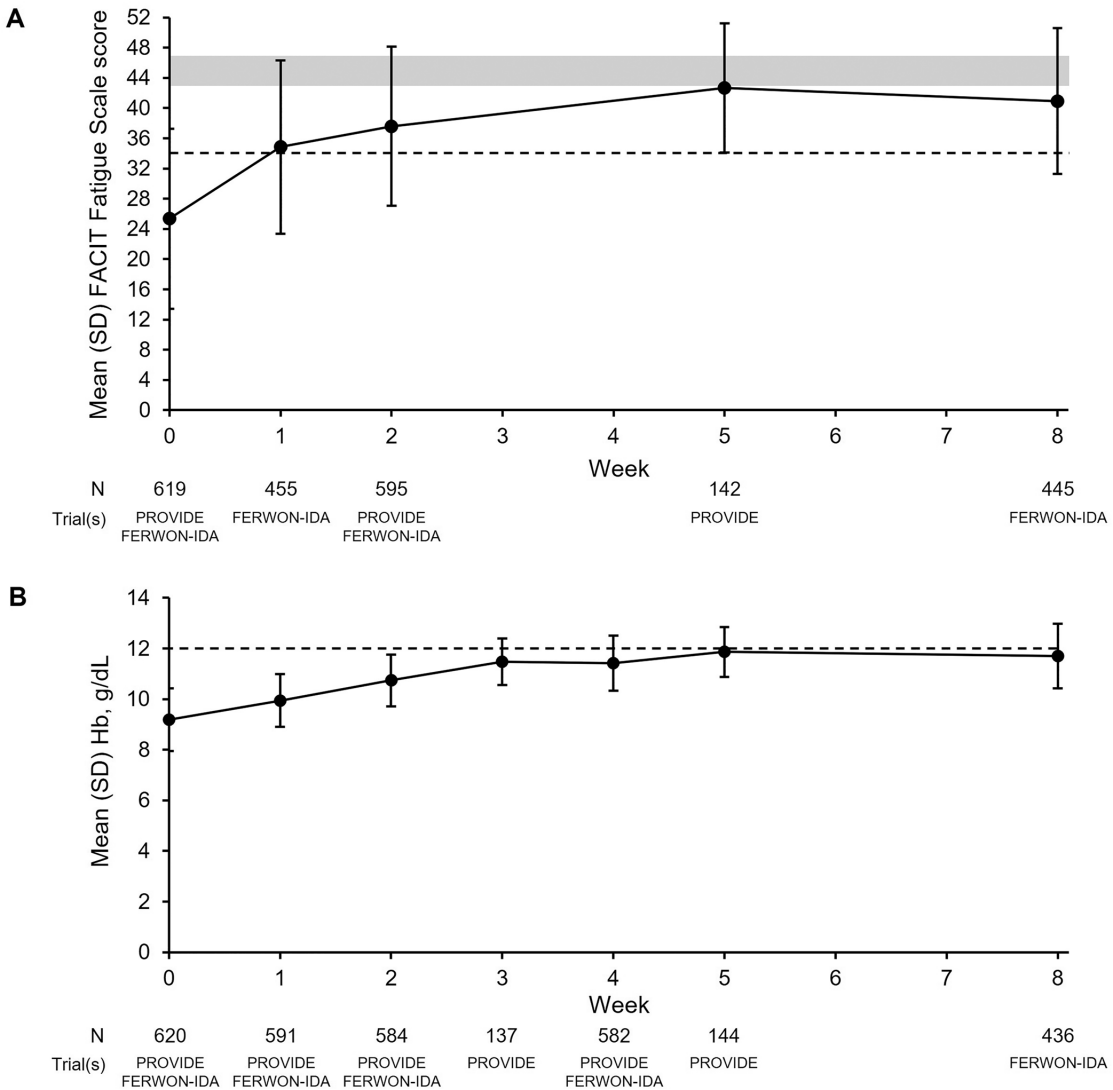
Mean FACIT Fatigue Scale score (A) and mean Hb (B) from baseline to Week 8 with FDI. Data are from the full analysis set. A higher score on the FACIT Fatigue Scale indicates lower levels of fatigue. The shaded area on panel A represents a score range within which United States general population norms for FACIT Fatigue Scale score (men and women) have been reported (mean scores of 43.6–46.6 points) in the literature (Butt Z et al. J Pain Symptom Manage. 2010;40(2):217–223, and Cella D et al. Cancer. 2002;94(2):528–538) – scores for women only were not identified. The dashed line on panel A (at 34 points) represents an estimated threshold for severe fatigue based on the literature (Piper BF, Cella D. J Natl Compr Canc Netw. 2010;8(8):958–966, and Eek D et al. J Patient Rep Outcomes. 2021;5(1):27). The dashed line on panel B represents the Hb cut‐off used (Hb < 12 g/dL) in the definition for IDA in this *post hoc* analysis. FACIT = Functional Assessment of Chronic Illness Therapy, FDI = ferric derisomaltose, Hb = hemoglobin, IDA = iron deficiency anemia, SD = standard deviation.

Overall, 75.2% of participants (*n* = 436/580) achieved a Hb level ≥ 12 g/dL or a rise in Hb of ≥ 2 g/dL, 81.6% (*n* = 480/588) had an increase of ≥ 5 points on the FACIT Fatigue Scale, and 61.6% (*n* = 362/588) had an increase of ≥ 12 points on the FACIT Fatigue Scale. Significantly more participants who had a hematological response (Hb ≥ 12 g/dL or a rise in Hb of ≥ 2 g/dL at the last study visit) experienced an improvement of ≥ 5 on the FACIT Fatigue Scale compared to those without a hematological response (84.4% [*n* = 368/436] vs. 72.2% [*n* = 104/144]; *p* < 0.01). Similarly, significantly more participants who had a hematological response (64.4%, *n* = 281/436) experienced an improvement of ≥ 12 on the FACIT Fatigue Scale compared to those without a hematological response (53.5%, *n* = 77/144; *p* < 0.05). These data are also shown in Appendix 3.

A total of 246 ADRs were reported in 19.6% of participants (*n* = 123/626) who received at least one dose of FDI (safety analysis set). Of the 246 ADRs, 242 ADRs were non‐serious. ADRs occurring in ≥ 2% of participants were nausea (*n* = 17/626; 2.7%) and rash (*n* = 18/626; 2.9%). Serious ADRs were reported in three participants (< 1%); of these participants, two experienced hypersensitivity, one experienced dyspnea, and one experienced a pruritic rash. No cases of anaphylaxis were reported as a serious ADR.

This *post hoc* analysis found that improvements in Hb correlated with reductions in fatigue in individuals with AUB. FDI was well tolerated, which aligns with the findings for the broader IDA populations in PROVIDE and FERWON‐IDA [3, 4]. While IS displays a similar efficacy and safety profile in the AUB population (analyses conducted for completeness; data not shown), FDI offers a dose advantage, as a higher amount can be given in fewer infusions. It is worth noting that a substantial number of participants (over 50%) treated with FDI who did not have a hematological response experienced an improvement in fatigue, regardless of the definition of fatigue response used. This is tangential evidence that it may be beneficial to treat ID even in the absence of anemia.

The results should be interpreted in the context of the study design. As PROVIDE and FERWON‐IDA were open‐label trials, the possible effect of patient bias cannot be excluded, particularly concerning the patient‐reported fatigue instrument used. The schedules for study visits differed between PROVIDE and FERWON‐IDA, so data for some time points were from one trial only. It was impossible to make corrections for multiple comparisons due to the *post hoc* nature of the study. Detailed patient history was not available to provide additional context (e.g., on prior intolerance or unresponsiveness to oral iron, or on concurrent treatment that could have confounded the results). There was also the potential for misclassification due to the definitions of the causes of AUB. However, analyses were repeated with and without the leiomyoma group to check for misclassification bias, with no difference in the results. Individuals may have had ongoing AUB, which could have led to a more conservative improvement in Hb levels and fatigue than if bleeding was resolved, and this is a factor that should be considered in the management of IDA. Additionally, the doses given in PROVIDE and FERWON‐IDA may not have been sufficient to adequately replenish Hb levels in all patients, some of whom may have required redosing, though this was not investigated by the trials.

In conclusion, this *post hoc* study found that FDI effectively reduced levels of fatigue, and was well tolerated, in a pooled population with AUB‐associated IDA.

## Ethics Statement

This was a *post hoc* analysis of data selected and pooled from two trials, PROVIDE and FERWON‐IDA; these trials were approved by relevant institutional review boards and conducted in accordance with good clinical practice and the Declaration of Helsinki, as revised in 2008, with informed consent obtained from all participants.

## Consent

The PROVIDE and FERWON‐IDA trials, from which select data were pooled for this *post hoc* analysis, obtained written informed consent from all participants.

## Conflicts of Interest

Petra Stute has received speaker fees from Pharmacosmos, Pierre Fabre, Besins Healthcare, Jenapharm, Theramex, and Exeltis, and has been a consultant for Astellas. Imo J. Akpan has been a consultant and speaker for Pharmacosmos, and has served on advisory boards for Pharmacosmos. Christian Breymann has given sponsored talks for Pharmacosmos and Pierre Fabre Switzerland. Ally Murji has participated in speaker bureaus and served on advisory boards for Abbvie, Bayer, Pfizer, and Pharmacosmos. Sarah H. O'Brien has been a consultant for Pharmacosmos and AstraZeneca. Jacquelyn M. Powers has been a consultant for Pharmacosmos. Malcolm G. Munro has been a consultant for American Regent, Daiichi Sankyo, Hologic Inc., Pharmacosmos, Shield Therapeutics, and Vifor.

## Supporting information


**Data S1.** Supporting Information.

## Data Availability

Research data are not shared.
