# Supplementary material for: Effect of Ferric Derisomaltose on Fatigue in Iron Deficiency Anemia Associated With Abnormal Uterine Bleeding
Source: Am J Hematol. 2024 Dec 12;100(3):497–500. doi: 10.1002/ajh.27555 (PMC11803534; doi:10.1002/ajh.27555)
Supplement: Supplementary file 1 — Data S1. Supporting Information. [file AJH-100-497-s001.pdf]

Supplemental Digital Content

Effect of ferric derisomaltose on fatigue in iron deficiency anemia associated with abnormal uterine bleeding

Authors

Petra Stute, MD, PhD,[1] Imo J. Akpan, MD,[2] Christian Breymann, MD, PhD,[3] Ally Murji, MD, MPH,[4] Sarah H. O'Brien, MD, MSc,[5] Jacquelyn M. Powers, MD, MS,[6,7] Malcolm G. Munro, MD[8]

Affiliations

1. Department of Obstetrics and Gynecology, Inselspital, Bern University Hospital, Bern, Switzerland

2. Division of Hematology and Oncology, Columbia University Irving Medical Center, New York, NY, USA

3. OBGYN Center Gyn-Perinatal Zürich, Hirslanden Clinics, Switzerland

4. Department of Obstetrics and Gynecology, Trillium Health Partners and The Institute for Better Health, University of Toronto, Toronto, Ontario, Canada

5. Division of Pediatric Hematology, Oncology, and Blood and Marrow Transplant, Nationwide Children's Hospital and The Ohio State University College of Medicine, Columbus, OH, USA

6. Department of Pediatrics, Division of Hematology/Oncology, Baylor College of Medicine, Texas Children's Cancer and Hematology Center, Houston, TX, USA

7. Department of Pediatrics, Baylor College of Medicine, Houston, TX, USA

8. Department of Obstetrics and Gynecology, David Geffen School of Medicine at UCLA, Los Angeles, CA, USA

Corresponding author

Petra Stute

Department of Obstetrics and Gynecology, Inselspital, Bern University Hospital, Bern, Switzerland.

E-mail address: Petra.Stute@insel.ch

Appendix 1. Flow diagram of participants from PROVIDE[1] and FERWON-IDA[2] included in the *post hoc* analysis

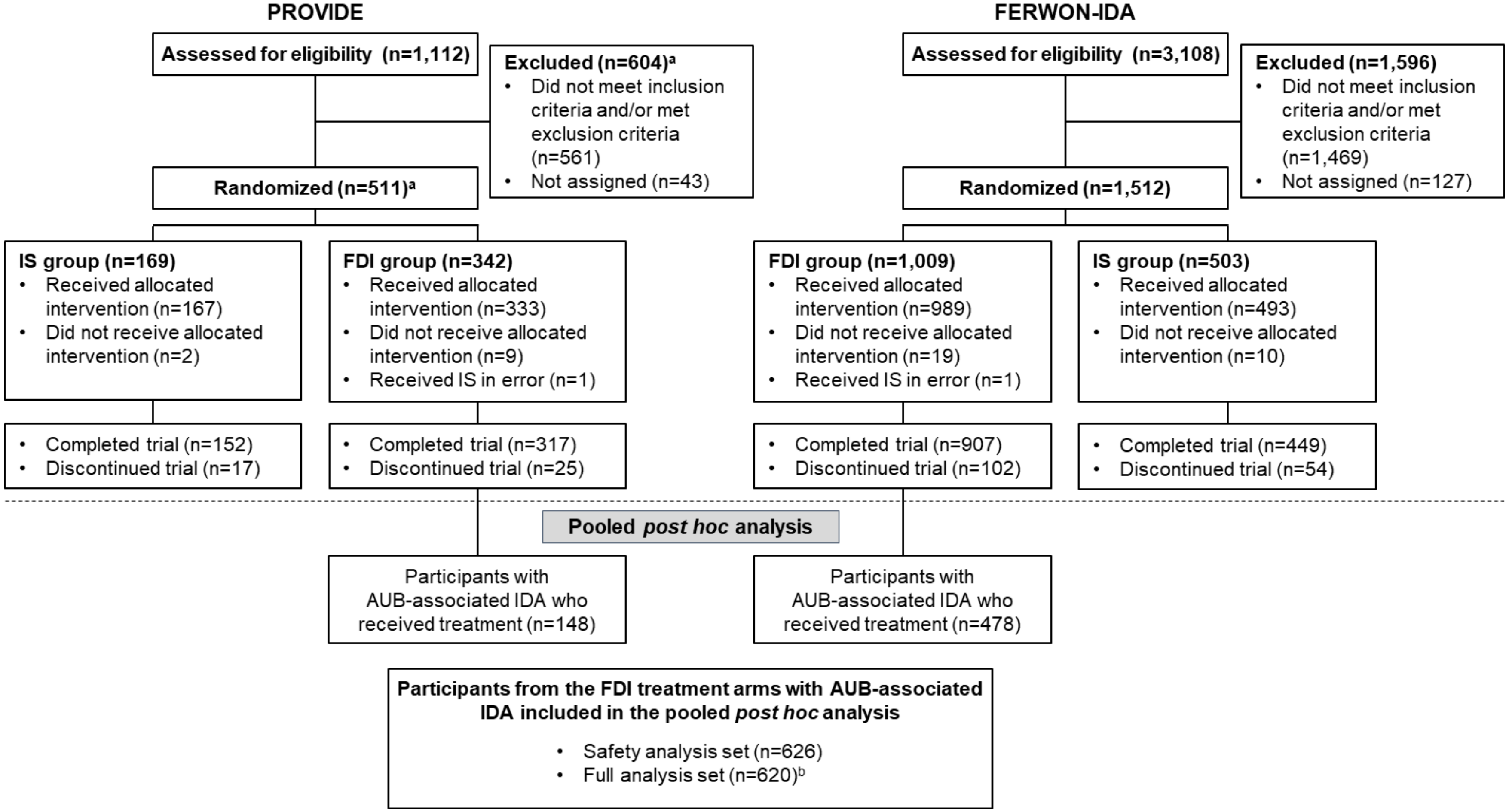

In PROVIDE, the cumulative dose of FDI (1,000 mg, 1,500 mg, or 2,000 mg) was given intravenously, either as a stand-alone dose of 1,000 mg or with an additional 500 mg or 1,000 mg dose one week later, depending on Hb level and body weight.[1] In FERWON-IDA, FDI was administered as a single IV dose of 1,000 mg at baseline.[2] [a]Three patients failed screening but were randomized in error – two patients in the FDI group were later withdrawn, and one patient completed the trial. [b]One participant included in the full analysis set had been randomized to FDI but received IS in error. IS=iron sucrose, FDI=ferric derisomaltose, AUB=abnormal uterine bleeding, IDA=iron deficiency anemia.

Note: this figure contains material adapted from the PROVIDE (Derman et al., 2017) and FERWON-IDA (Auerbach et al., 2019) articles published in Am J Hematol, used under the Creative Commons Attribution (CC BY 4.0) license (https://creativecommons.org/licenses/by/4.0/). Changes include editing, formatting, and the addition of original content. Source materials are available at https://doi.org/10.1002/ajh.24633 [PROVIDE] and https://doi.org/10.1002/ajh.25564 [FERWON-IDA].

Appendix 2. Baseline demographics and clinical characteristics of the ferric derisomaltose population

Characteristic	Participants (N=620)
Age (years), mean (SD) [range]	38.4 (8.5) [18–55]
Age group, n (%)	
18–≤35 years	208 (33.5)
>35–≤40 years	118 (19.0)
>40–≤45 years	149 (24.0)
>45–≤50 years	117 (18.9)
>50–≤55 years	28 (4.5)
Race,[a] n (%)	
Asian	7 (1.1)
Black or African American	343 (55.3)
White	255 (41.1)
None of the above	15 (2.4)
Weight (kg), mean (SD) [range]	86.5 (24.0) [39–209]
BMI (kg/m^2), mean (SD) [range]	32.1 (8.5) [14–71][b]
Current active smoker, n (%)	77 (12.4)
Hb (g/dL), mean (SD) [range]	9.2 (1.2) [4.4–11.8]
Proportion of participants by severity of anemia, n (%)	
Mild-to-moderate anemia (≥9–<12 g/dL)	367 (59.2)
Severe anemia (Hb <9 g/dL)	253 (40.8)
Serum ferritin (ng/mL), mean (SD) [range]	8.1 (9.7) [0.5–88.0]
TSAT (%), mean (SD) [range]	5.6 (3.3) [1.0–19.0]
FACIT Fatigue Scale score, mean (SD) [range]	25.4 (11.9) [0.0–52.0][b]

Data are from the full analysis set. There were no malignant neoplasms in the study population. [a]Participants were asked by a healthcare professional what race best describes them from select options available in a validated electronic case report: 'American Indian or Alaskan Native', 'Asian', 'Black or African American', 'Native Hawaiian or other Pacific Islander', 'White', or 'Other'. [b]n=619. BMI=body mass index, FACIT=Functional Assessment of Chronic Illness Therapy, Hb=hemoglobin, SD=standard deviation, TSAT=transferrin saturation.

Appendix 3. Proportion of participants with a hematological and/or fatigue response after treatment with FDI

	Participants	P-value
Proportion of participants with a hematological response, n/N (%)		
Hb ≥12 g/dL or a rise in Hb of ≥2 g/dL	436/580 (75.2)	–
Proportion of participants with a fatigue response, n/N (%)		
Improvement of ≥5 on FACIT Fatigue Scale	480/588 (81.6)	–
Improvement of ≥12 on FACIT Fatigue Scale	362/588 (61.6)	–
Proportion of participants with a fatigue response according to presence/absence of hematological response, n/N (%)		
Improvement of ≥5 on FACIT Fatigue Scale		
In participants with a hematological response	368/436 (84.4)	<0.01
In participants without a hematological response	104/144 (72.2)	
Improvement of ≥12 on FACIT Fatigue Scale		
In participants with a hematological response	281/436 (64.4)	<0.05
In participants without a hematological response	77/144 (53.5)	

Observed case data were used, with no missing data imputation. P-values were derived from a logistic regression model with treatment and hematological subgroup (i.e., participants with or without a hematological response) as factors and with an interaction between treatment and hematological subgroup.

FACIT=Functional Assessment of Chronic Illness Therapy, FDI=ferric derisomaltose; Hb=hemoglobin.

References

1. Derman R, Roman E, Modiano MR, et al. A randomized trial of iron isomaltoside versus iron sucrose in patients with iron deficiency anemia. *Am J Hematol*. 2017;92(3):286-291. doi:10.1002/ajh.24633

2. Auerbach M, Henry D, Derman RJ, et al. A prospective, multi-center, randomized comparison of iron isomaltoside 1000 versus iron sucrose in patients with iron deficiency anemia; the FERWON-IDA trial. *Am J Hematol*. 2019;94(9):1007-1014. doi:10.1002/ajh.25564
